# P-1382. Stress hyperglycemia during tuberculosis treatment and rate of sputum culture conversion

**DOI:** 10.1093/ofid/ofaf695.1569

**Published:** 2026-01-11

**Authors:** Fay Willis, Maia Kipiani, Dzigbordi Kamasa-Quashie, Tekla Madzgharashvili, Inga Davitadze, Victoria Ontiveros, Hardy Kornfeld, Lorissa Smulan, Francisco J Pasquel, Russell R Kempker, Jeffrey M Collins, A Nichole Evans, Angie Campbell, Neel Gandhi, Matthew J Magee

**Affiliations:** Emory University, Rollins School of Public Health, Department of Epidemiology, Chattanooga, TN; National Center for Tuberculosis and Lung Diseases, Tbilisi, Tbilisi, Georgia; Emory University Rollins School of Public Health, Atlanta, Georgia; National Center for Tuberculosis and Lung Diseases, Tbilisi, Tbilisi, Georgia; Salikh Abashidze's Regional Center for Infectious Diseases, Aids and Tuberculosis, Batumi, Georgia, Batumi, Ajaria, Georgia; Emory University Rollins School of Public Health, Atlanta, Georgia; Department of Medicine, University of Massachusetts Chan Medical School, Worcester, Massachusetts; UMass Chan Medical School, Worcester, Massachusetts; Emory, University School of Medicine, Atlanta, Georgia; Emory University School of Medicine, Atlanta, GA; Emory University School of Medicine, Atlanta, GA; Emory University Rollins School of Public Health, Atlanta, Georgia; Rollins School of Public Health, Atlanta, Georgia; Rollins School of Public Health, Atlanta, Georgia; Emory University, Atlanta, Georgia

## Abstract

**Background:**

Tuberculosis (TB) is the leading cause of infectious disease mortality worldwide. Diabetes increases the risk of poor TB treatment outcomes and is increasingly prevalent in high-TB burden settings. However, whether TB-induced stress hyperglycemia impacts TB treatment outcomes is unclear. We compared TB culture conversion rates among stress hyperglycemic participants to those with diabetes/pre-diabetes or euglycemia.

**Methods:**

This is an on-going prospective cohort study of adults with newly-diagnosed pulmonary TB disease in the country of Georgia. At treatment initiation, participants were categorized as having diabetes (DM)/prediabetes (pre-DM), euglycemia, or stress hyperglycemia (defined as hemoglobin A1c [HbA1c] ≥ 5.7% or fasting blood glucose [FBG] ≥ 100 mg/dl at TB treatment start and HbA1c < 5.7% or FBG < 100 mg/dl within 2 months). Culture conversion was defined as two consecutive monthly negative *Mtb* cultures with no subsequent positive cultures. Culture conversion rates were compared by glycemic status using Kaplan Meier curves and hazards regression.

**Results:**

Among 225 participants (64% of target enrollment) with culture data available to date: median age was 40 years (IQR 29-54), median BMI was 20.7 kg/m^2^ (IQR 18.6-23.8), and 38% were female. At enrollment, 43% had stress hyperglycemia, 17% had DM or pre-DM, and 40% had euglycemia. Overall, 200 participants (89%) achieved culture conversion by 2 months, with the lowest proportion of culture conversion in the stress hyperglycemia group (82% converted, vs 89% DM/pre-DM and 96% euglycemic; p=0.01). Median time to culture conversion was 58 days (IQR 30-62) among participants with stress hyperglycemia, compared to 59 days (IQR 31-72) among DM/pre-DM and 51 days (IQR 30-62) among euglycemic participants (Figure 1; p=0.10). After adjusting for age, sex, and baseline BMI, participants with stress hyperglycemia (HR 0.75, 95%CI 0.5-1.0) and DM/pre-DM (HR 0.72, 95%CI 0.5-1.1) had reduced culture conversion rates compared to euglycemic participants.

**Conclusion:**

In preliminary analysis, we found >40% of participants had stress hyperglycemia at TB treatment initiation. Participants with stress hyperglycemia trended to have reduced culture conversion rates compared to those with euglycemia.

**Disclosures:**

Francisco J. Pasquel, MD, MPH, Dexcom: Grant/Research Support|Dexcom: Grant/Research Support|Dexcom: Honoraria|Ideal Medical Technologies: Grant/Research Support|Insulet: Grant/Research Support|Novo Nordisk: Grant/Research Support|Tandem Diabetes Care: Grant/Research Support

